# The Symptom Monitoring with Feedback Trial (SWIFT): protocol for a registry-based cluster randomised controlled trial in haemodialysis

**DOI:** 10.1186/s13063-022-06355-0

**Published:** 2022-05-19

**Authors:** Lavern Greenham, Paul N. Bennett, Kathryn Dansie, Andrea K. Viecelli, Shilpanjali Jesudason, Rebecca Mister, Brendan Smyth, Portia Westall, Samuel Herzog, Chris Brown, William Handke, Suetonia C. Palmer, Fergus J. Caskey, Cecile Couchoud, John Simes, Stephen P. McDonald, Rachael L. Morton

**Affiliations:** 1 Australia and New Zealand Dialysis and Transplant Registry, Adelaide, SA Australia; 2 Satellite Healthcare, San Jose, CA USA; 3 University of South Australia, Adelaide, SA Australia; 4 Princess Alexandra Hospital, Woolloongabba, QLD Australia; 5 Faculty of Medicine, University of Queensland, Brisbane, Australia; 6 Central Northern Adelaide Renal and Transplantation Service, Royal Adelaide Hospital, Adelaide, SA Australia; 7 University of Adelaide, Adelaide, SA Australia; 8 NHMRC Clinical Trials Centre, University of Sydney, Camperdown, NSW Australia; 9 Department of Renal Medicine, St George Hospital, Kogarah, NSW Australia; 10 Consumer Representative, Canberra, ACT Australia; 11 University of Otago, Canterbury, Christchurch New Zealand; 12 University of Bristol, Bristol, UK; 13 Renal Epidemiology and Information Network (REIN), Agence de la Biomédecine, Saint-Denis, Paris, France

**Keywords:** Kidney replacement therapy, Renal dialysis, Haemodialysis, Patient-reported outcomes, Chronic kidney disease, Quality of life, Registry, Cluster and randomised controlled trial

## Abstract

**Background:**

Kidney failure prevalence is increasing worldwide. Haemodialysis, peritoneal dialysis or kidney transplantation are undertaken to extend life with kidney failure. People receiving haemodialysis commonly experience fatigue, pain, nausea, cramping, itching, sleeping difficulties, anxiety and depression. This symptom burden contributes to poor health-related quality of life (QOL) and is a major reason for treatment withdrawal and death. The Symptom monitoring WIth Feedback Trial (SWIFT) will test the hypothesis that regular symptom monitoring with feedback to people receiving haemodialysis and their treating clinical team can improve QOL.

**Methods:**

We are conducting an Australia and New Zealand Dialysis and Transplant (ANZDATA) registry-based cluster randomised controlled trial to determine the clinical- and cost-effectiveness at 12 months, of 3-monthly symptom monitoring using the Integrated Palliative Outcome Scale-Renal (IPOS-Renal) survey with clinician feedback, compared with usual care among adults treated with haemodialysis. Participants complete symptom scoring using a tablet, which are provided to participants and to clinicians. The trial aims to recruit 143 satellite haemodialysis centres, (up to 2400 participants). The primary outcome is change in health-related QOL, as measured by EuroQol 5-Dimension, 5-Level (EQ-5D-5L) instrument. Secondary outcomes include overall survival, symptom severity (including haemodialysis-associated fatigue), healthcare utilisation and cost-effectiveness.

**Discussion:**

SWIFT is the first registry-based trial in the Australian haemodialysis population to investigate whether regular symptom monitoring with feedback to participants and clinicians improves QOL. SWIFT is embedded in the ANZDATA Registry facilitating pragmatic recruitment from public and private dialysis clinics, throughout Australia. SWIFT will inform future collection, storage and reporting of patient-reported outcome measures (PROMs) within a clinical quality registry. As the first trial to rigorously estimate the efficacy and cost-effectiveness of routine PROMs collection and reporting in haemodialysis units, SWIFT will provide invaluable information to health services, clinicians and researchers working to improve the lives of those with kidney failure.

**Trial registration:**

Australian New Zealand Clinical Trials Registry ACTRN12620001061921. Registered on 16 October 2020

**Supplementary Information:**

The online version contains supplementary material available at 10.1186/s13063-022-06355-0.

## Administrative information

Note: the numbers in curly brackets in this protocol refer to SPIRIT checklist item numbers. The order of the items has been modified to group similar items (see http://www.equator-network.org/reporting-guidelines/spirit-2727-statement-defining-standard-protocol-items-for-clinical-trials/).

**Table:** 

Title {1}	The Symptom Monitoring with Feedback Trial (SWIFT): Protocol for a registry-based cluster randomised controlled trial in haemodialysis
Trial registration {2a and 2b}.	Australian New Zealand Clinical Trials Registry #ACTRN12620001061921 Prospectively registered 16^th^ October 2020
Protocol version {3}	Version 3.0 14^th^ December 2020
Funding {4}	Australian NHMRC Project Grant #1159051; KHA Project Grant KHA2018-RM; NHMRC TRIP Fellowship #1150989 RM; BEAT-CKD NHMRC Program Grant #1159051, NHMRC Investigator Grant #1196033, Queensland Advancing Clinical Research Fellowship Grant.
Author details {5a}	Lavern M. Greenham^1^, Paul N. Bennett^2,3^, Kathryn Dansie^1^, Andrea K. Viecelli^4,5^, Shilpanjali Jesudason^6,7^, Rebecca Mister^8^, Brendan Smyth^8,9^, Portia Westall^8^, Sam Herzog^8^, Chris Brown^8^, William Handke^10^, Suetonia C. Palmer^11^, Fergus J. Caskey^12^, Cecile Couchoud^13^, John Simes^8^, Stephen P. McDonald^1,6,7^, Rachael L. Morton^8*^ ^1^Australia and New Zealand Dialysis and Transplant Registry, Adelaide, SA, Australia. ^2^Satellite Healthcare, San Jose, CA, USA. ^3^University of South Australia, Adelaide, SA, Australia. ^4^Princess Alexandra Hospital, Woolloongabba, QLD, Australia. ^5^Faculty of Medicine, University of Queensland, Brisbane, Australia. ^6^Central Northern Adelaide Renal and Transplantation Service, Royal Adelaide Hospital, Adelaide, SA, Australia. ^7^University of Adelaide, Adelaide, SA, Australia. ^8^NHMRC Clinical Trials Centre, University of Sydney, Camperdown, NSW, Australia. ^9^Department of Renal Medicine, St George Hospital, Kogarah, NSW, Australia. ^10^Consumer Representative, Canberra, ACT, Australia. ^11^University of Otago, Christchurch, Canterbury, New Zealand. ^12^University of Bristol, Bristol, UK. ^13^Renal Epidemiology and Information Network (REIN), Agence de la Biomédecine, Saint-Denis, Paris, France.
Name and contact information for the trial sponsor {5b}	University of Sydney, National Health and Medical Research Council Clinical Trials Centre (NHMRC CTC), Levels 4-6 Medical Foundation Building, 92-94 Parramatta Rd, Camperdown NSW 2050
Role of sponsor {5c}	The study sponsor and the study funders (Australian NHMRC, Kidney Health Australia), did not have any role or ultimate authority in study design; collection, management, analysis, interpretation of data, writing of the report, or the decision to submit the report for publication.

## Introduction

### Background and rationale {6a}

Kidney disease, consisting of chronic kidney disease (CKD), acute kidney injury (AKI) and kidney failure, affects 850 million people globally [1–3]. People with kidney failure can receive kidney replacement therapy (KRT) in the form of either dialysis or kidney transplantation to extend life. It is predicted that by the year 2030, the need for KRT will reach approximately 5.4 million people, the majority in low- and middle-income countries [4]. Dialysis, the most common KRT worldwide, has significant costs yet results in only 51% survival at 5 years [5], lower than all cancers combined [6]. In Australia in 2020, 13,931 people were receiving dialysis; 10,470 (75%) in haemodialysis facilities (non-hospital or hospital) with 9% undertaking haemodialysis at home and a further 16% performing peritoneal dialysis [7].

Facility-based haemodialysis typically requires three treatment sessions per week for 3 to 5 h per session. This treatment removes solutes and fluid directly from the blood; however, people receiving this therapy frequently report symptoms of fatigue, pain, nausea, cramping, hypotension, itching, sleeping difficulties, anxiety and depression [8]. This is reflected in the low quality of life (QOL) of people receiving facility-based haemodialysis, with self-reported QOL at 59% of full health [9]. This is lower than the self-reported QOL of people with metastatic prostate cancer or spinal cord injury [10].

Overwhelming symptoms and poor QOL can lead to dialysis withdrawal and death. In 2020, the ANZDATA Registry reported 575 deaths (34% of all haemodialysis deaths) attributed to withdrawal of haemodialysis [11]. Of these, 202 (35%) were due to psychosocial reasons, including symptom burden and poor QOL [11]. More frequent or longer dialysis sessions may reduce some symptoms; however, this strategy may only result in small improvements in QOL [12]. Symptom management improves QOL and survival in other disease conditions. In cancer care, symptom monitoring during routine chemotherapy improved survival compared with usual care [13]. Symptom management in palliative care improved QOL, reduced symptoms and increased functional wellbeing compared with standard care [14]. The use of patient-reported outcome measures (PROMs) including symptom monitoring is recommended to guide clinical care for many conditions; however, few trials have assessed the effectiveness of this intervention [1]. In haemodialysis populations, although effective targeted management of symptoms is associated with improved QOL [15], evidence is lacking from randomised controlled trials (RCT).

In 2020, we completed a SWIFT pilot study among four dialysis units in South Australia and Queensland [16, 17]. Both patients and clinicians found the tablet computers easy to use and felt that electronic collection of PROMs was useful and important for patients’ QOL. The pilot concluded that electronic symptom monitoring in adults on haemodialysis with feedback to clinicians was feasible [17]. Lessons learned from the pilot have shaped the design of the main trial.

### Objectives {7}

SWIFT evaluates the hypothesis that regular symptom monitoring with feedback to people receiving haemodialysis and their clinicians improves QOL at 12 months. SWIFT is a registry-based cluster RCT to determine the clinical- and cost-effectiveness of 3-monthly symptom monitoring using the validated Integrated Palliative Outcome Scale – Renal (IPOS-Renal) survey. The intervention includes symptom score feedback to adult haemodialysis participants and clinicians with links to evidence-based symptom management also sent to clinicians. Secondary outcomes include survival, frequency and severity of symptoms (including haemodialysis-associated fatigue), biochemical measures of dialysis adequacy, dialysis duration and frequency, dialysis withdrawal and healthcare utilisation. In addition, the trial is investigating whether electronic capture of patient-reported outcomes within a clinical quality registry at a national level is feasible and cost-effective.

### Trial design {8}

**Fig. 1:**
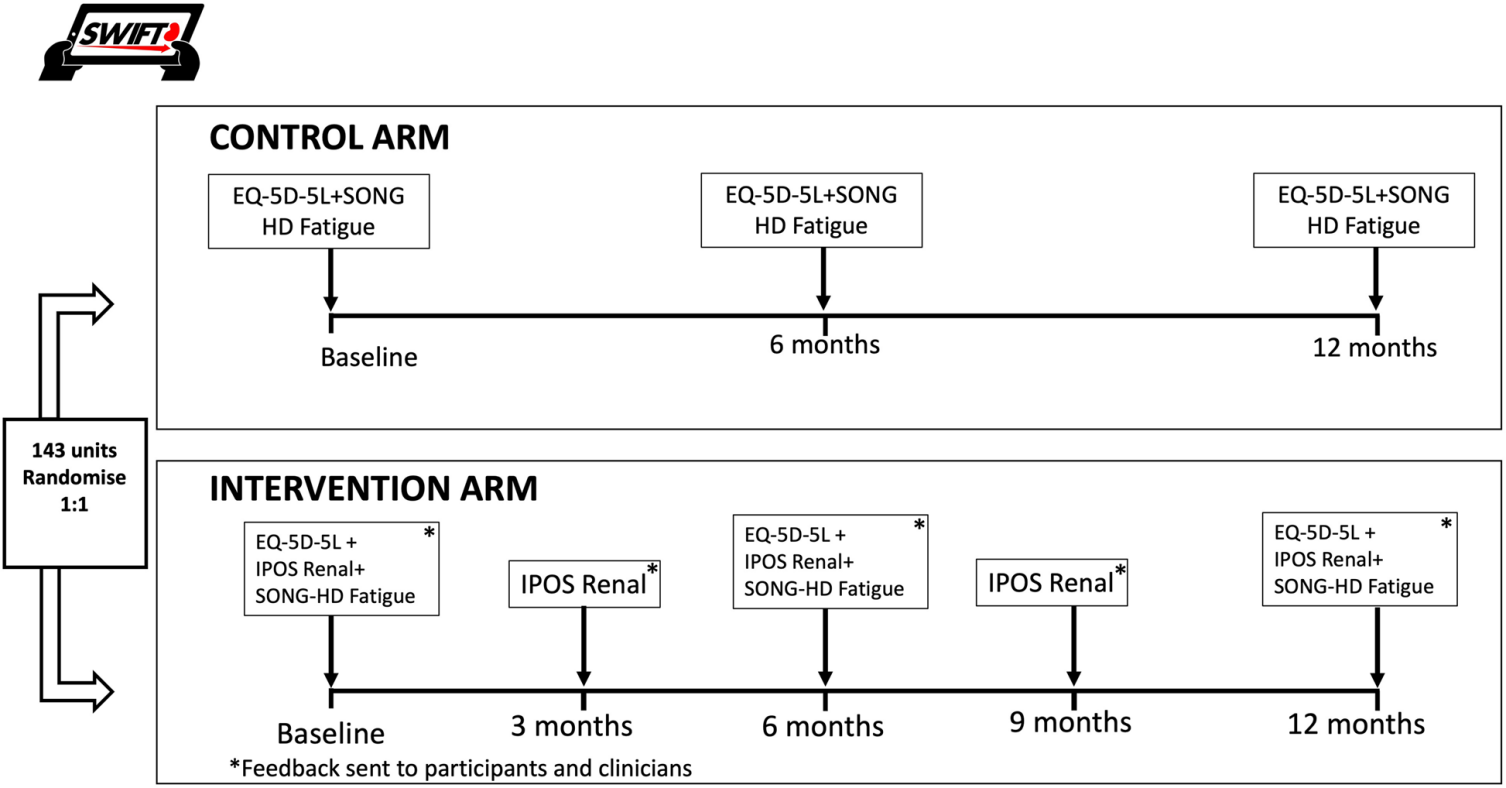
Trial schema for SWIFT

The trial is a prospective registry-based cluster randomised controlled trial of 3-monthly electronic symptom collection using IPOS-Renal with feedback to clinicians and participants versus usual care for adults managed with centre haemodialysis (Fig. 1). This trial followed the Standard Protocol Items: Recommendations for Interventional Trials (SPIRIT) guideline [18] (see Supplementary Material). Haemodialysis centres (the clusters) will be randomised in a 1:1 ratio with linked parent/satellite centres forced to the same allocated intervention to account for patient movement between centres. The trial will be conducted and reported according to the CONSORT 2010 checklist for reporting a cluster randomised trial [15] and patient-reported outcomes in trials [16]. The CHEERS guidelines will guide reporting of cost-effectiveness [19].

## Methods: participants, interventions and outcomes

### Study setting {9}

SWIFT is being conducted in both public and private haemodialysis centres across Australia. These are centres that are predominantly nurse-managed with visiting on-site physician attendance [20]. Haemodialysis is covered by publicly funded healthcare coverage and is primarily provided by state-run health networks. Some networks have contracted private providers for some or all satellite dialysis services. Regardless of provider, all dialysis care is free to the patient [21], and all dialysis units send patient data to the ANZDATA Registry.

### Eligibility criteria {10}

#### Inclusion criteria

All centres contributing to the ANZDATA Registry (a bi-national registry which covers all chronic dialysis providers) with a minimum of 10 patients are eligible for participation. Within these centres, adults aged 18 years and older, who are willing and able to adhere to all trial requirements and able to provide informed consent, will be invited to participate. In order to encourage participation of those from a culturally and linguistically diverse background, SWIFT has translated survey materials for adults who read simplified Chinese, traditional Chinese, Vietnamese, Korean, Arabic, Greek or Italian text. Centres currently using the IPOS-Renal questionnaire for symptom assessment are still eligible to participate if they agree to use the tablet-based SWIFT data collection and follow the protocol as per their allocation during the trial period.

#### Exclusion criteria

People under 18 years of age or unable to provide informed consent are excluded. In addition, those people or centres who are participating in programmes or trials in which an extensive electronic symptom monitoring and feedback management system is already in place (where the centre or individual is not willing to pause current management, and where co-enrolment is unfeasible) are excluded.

### Who will take informed consent? {26a}

The nurse champion or their delegate (other nursing staff, allied health professionals, clinical research assistants or other nominated persons) in each dialysis centre will assist with recruitment and consent by distributing the patient information sheets prior to survey completion and are available to answer trial-related questions from participants. The nurse champion takes responsibility for conducting the trial in their unit and is responsible for leading participant recruitment. Consent is obtained electronically from each participant prior to commencing the baseline survey.

### Additional consent provisions for collection and use of participant data and biological specimens {26b}

Additional consent will be sought from Australian participants for access to their hospitalisations and Medicare Benefits Schedule (MBS) and Pharmaceutical Benefits Scheme (PBS) records through registry data linkage. No biological specimens will be collected.

### Interventions

#### Explanation for the choice of comparators {6b}

As SWIFT is a pragmatic trial, usual care in the dialysis unit (the control arm) is the comparison to symptom monitoring and feedback. Participants in the control arm will not complete electronic 3-monthly IPOS-Renal measures, and the participants and clinicians in the control arm will not receive symptom scores with evidence-based recommendations for their management. Both arms will collect health-related quality of life through the EQ-5D-5L instrument, and these scores will not be fed back to clinicians or participants. Usual care will consist of the dialysis centre’s standard practice for measuring or monitoring symptoms and may constitute no symptom monitoring, ad hoc monitoring and monitoring using other mechanisms, (e.g. questions in a nursing assessment when a dialysis session is commenced, or medical history within a nephrology consultation). The ANZDATA audit of PROMs in haemodialysis centres revealed only around 12% of centres used symptom measures in a routine and systematic way during haemodialysis care [17, 22].

#### Intervention description {11a}

The intervention comprises regular measurement of symptom burden with feedback of these results to participants and clinicians. Participants in the intervention arm will complete the IPOS-Renal at baseline, 3 months, 6 months, 9 months and 12 months, within a 2-week window (Table 1). The individual participant’s IPOS-Renal results will be emailed to the centre nurse unit manager or delegate, and the participant’s treating nephrologist. Within the email will be a link to evidence-based guidelines for symptom management. The evidence-based guidelines were created by SWIFT Investigators (RLM and AKV) and are updated regularly (SWIFT Investigator BS) to ensure new evidence is incorporated. Any symptom score of 3 (severe symptoms) or 4 (overwhelming symptoms) on a scale of 0–4, on the IPOS-Renal instrument will be flagged for prompting clinical review. All subsequent medical treatments are at the discretion of the treating clinicians. Participants receive a copy of the completed survey emailed to them in real-time if they provide an email address at commencement of the survey. Based on our previous work, we anticipate about 30–40% of participants will provide an email address [17]. If a participant does not have their own email address, they can nominate the address of a family member or close friend. Participants do not receive the evidence-based guidelines for symptom management.

**Table 1:** Study timeline schedule

	Patient screening	Baseline	3 months	6 months	9 months	12 months
Patient demographics	X					
Informed consent		X				
EQ-5D-5L questionnaire		X		X		X
SONG-HD Fatigue questionnaire		X		X		X
IPOS Renal (*intervention arm only*)		X	X	X	X	X
Validity questions (all participants)		X		X		X
ANZDATA clinical treatment record	X	X	X	X	X	X
MBS and PBS data						X

*Abbreviations: EQ-5D-5L*, EuroQoL 5-item 5-level preference-based measure of health status, *IPOS Renal*, Integrated Palliative Outcome Scale – Renal, *SONG-HD Fatigue*, Standardised Outcomes in Nephrology – Haemodialysis, Fatigue

The IPOS-Renal is a 15-symptom checklist measures self-reported pain, shortness of breath, weakness, nausea, vomiting, poor appetite, constipation, sore mouth, drowsiness, poor mobility, itching, difficulty sleeping, restless legs, skin changes and diarrhoea. All symptom scores are reported on a 0 to 4 scale (0=not at all, 1=slightly, 2=moderately, 3=severely, 4=overwhelmingly bothered) and indicate the effect of the symptom on the respondent over the past week [23]. The IPOS-Renal takes approximately 9 min to complete and is widely used in palliative care [17]. Its use is recommended in the Australia and New Zealand Renal Supportive Care Guidelines [24].

#### Criteria for discontinuing or modifying allocated interventions {11b}

**Fig. 2:**
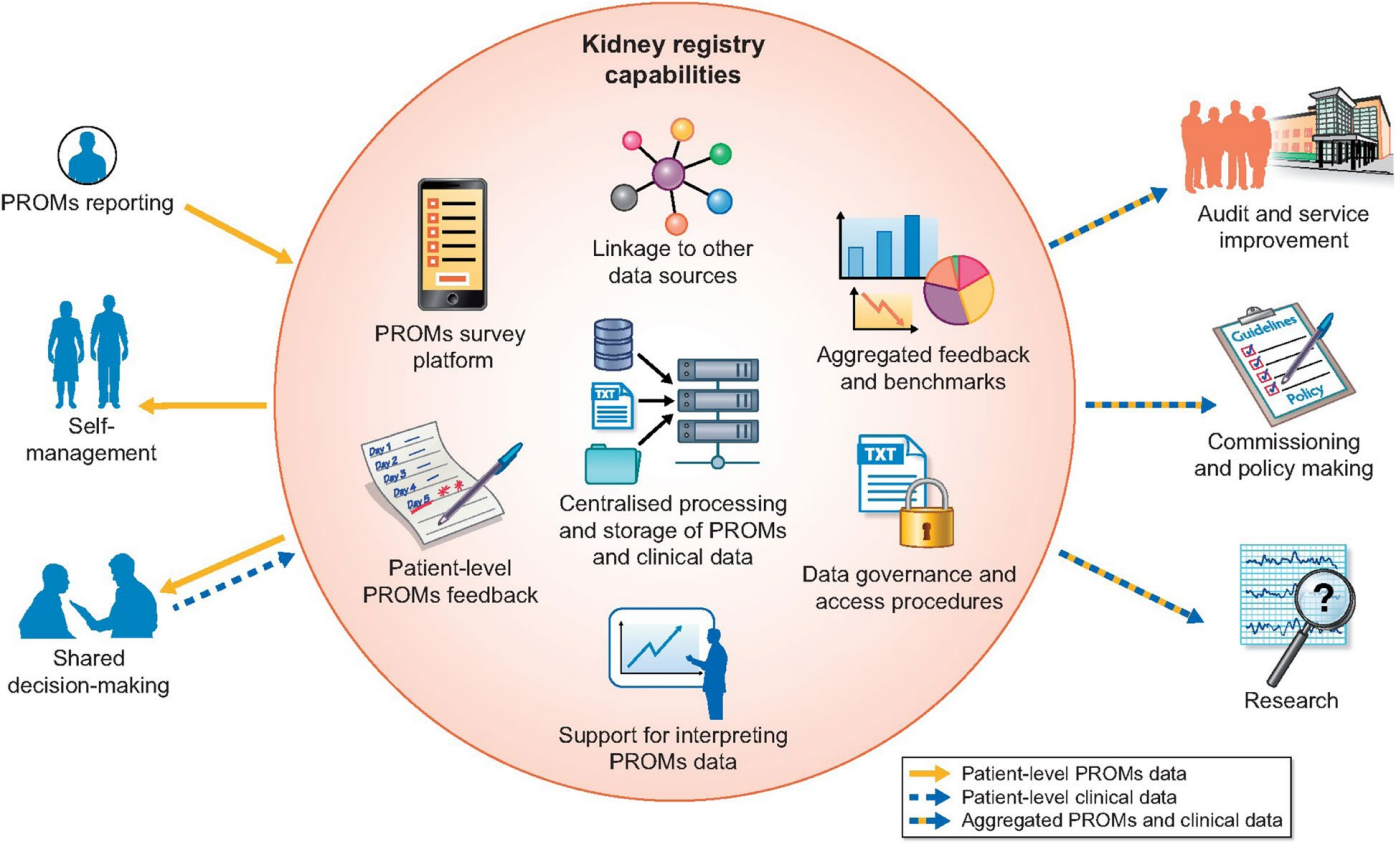
How kidney registries could use their capabilities to provide an infrastructure for facilitating large-scale collection of PROMs to support individual patient management and other purposes for multiple stakeholders

The trial intervention will be permanently discontinued if the participant declines further collection of PROMs, withdraws their consent to participate in the trial or ceases haemodialysis (i.e. receives a kidney transplant; is transferred to peritoneal dialysis, home haemodialysis or to a non-participating haemodialysis centre). Secondary outcomes will continue to be collected in the ANZDATA registry (see Fig. 2) and through linked administrative data unless consent for these data is also withdrawn.

#### Strategies to improve adherence to interventions {11c}

The SWIFT ANZDATA staff member will contact the SWIFT delegate in the unit (usually the nurse unit manager or ANZDATA contact) to compare the list of patients in the unit with those listed in the ANZDATA registry such that any discrepancies can be reconciled. This ensures that only patients who are in the unit at the time of recruitment and eligible for the trial are invited to participate. Each centre will appoint their own SWIFT nurse champion and will be assigned a research assistant (RA) from the SWIFT team. The RA will liaise with the SWIFT delegate in the unit during the trial to assist the coordination of the intervention. Where possible, the RA will attend the centre at the beginning of the trial recruitment phase. The RA will assist with logistical aspects such as site Wi-Fi connectivity and tablet functionality. These strategies to maintain engagement were adopted following consumer consultation in the SWIFT pilot study [16].

#### Relevant concomitant care permitted or prohibited during the trial {11d}

There are no restrictions on concomitant patient care during the trial. If a unit currently uses IPOS-Renal in some capacity, then this will be documented (stratification factor). The unit can be allocated to the control arm (symptom monitoring or feedback), and usual care will continue. If the unit is an IPOS-Renal user and is allocated to the intervention arm, SWIFT will request that the unit use the SWIFT monitoring intervention during the trial period. Units will have access to their patients’ IPOS-Renal data through the feedback and can request EQ-5D-5L data once the trial is complete.

#### Provisions for post-trial care {30}

There are no post-trial care requirements.

### Outcomes {12}

The primary outcome of SWIFT is the change in health-related QOL, as measured by EQ-5D-5L and compared between groups (Table 2). We also hypothesise that symptom monitoring and management may reduce overall and cause-specific mortality (including withdrawal from dialysis). In Australia in 2020, 575 of 1667 (34%) of dialysis deaths were due to withdrawal from dialysis. Of these, 202 (35%) of withdrawals were linked to psycho-social reasons [25]. It is anticipated that close monitoring of symptoms, discussions with patients about management of symptoms and therapeutic multi-disciplinary care aimed at reducing symptom burden may specifically reduce deaths due to withdrawal from dialysis. Secondary outcomes are listed in Table 2. Routine collection of secondary end points through the ANZDATA Registry already exists in an “opt-out” framework (#HREC/17/RAH/408).

**Table 2:** Table of SWIFT outcomes

Outcome	Description and unit of measure	Data collection timepoint or registry source
***Primary outcome***		
Health-related quality of life	Mean change in EQ-5D-5L value (utility) from baseline to 12 months (primary outcome)	Baseline, 6 months, 12 months
***Secondary outcome***		
Dialysis withdrawal	Number of participants identified as withdrawal from dialysis	12 months—collected through ANZDATA
Health-related quality of life	Mean change in EQ-5D-5L value (utility) from baseline to 6 months	Baseline, 6 months
Health-related quality of life	Mean change in EQ-5D-5L and VAS scores from baseline at 6 months; and from baseline to 12 months	Baseline, 6 months, 12 months
Overall survival	All-cause mortality rates at 12 months	12 months
Cause-specific mortality (including deaths due to dialysis withdrawal)	Cause-specific mortality rates at 12 months for dialysis withdrawal, cardiovascular, cancer, infection and other causes	12 months
Fatigue	Mean change in SONG-HD Fatigue score at 12 months	Baseline, 6 months, 12 months
Symptom severity	Intervention arm only. Measured by change in IPOS-Renal symptom severity scores	Baseline, 3 months, 6 months, 9 months, 12 months
Haemodialysis duration	Average number of hours per treatment	12 months—collected through ANZDATA
Haemodialysis frequency	Number of treatments per week	12 months—collected through ANZDATA
Haemodialysis adequacy	Urea reduction ratio and Kt/V	12 months—collected through ANZDATA
Symptom-related and general healthcare utilisation	Hospitalisations, Medicare Benefits Schedule (MBS), Pharmaceutical Benefits Scheme (PBS) items	Assessed through linked administrative records for admitted patient data and Medicare claims
Cost-effectiveness	Incremental cost per quality-adjusted life year (QALY) gained reported as an incremental cost effectiveness ratio (ICER) or incremental net benefit (INB)	12 months; EQ-5D-5L utilities collected as per primary outcome; healthcare use identified from linked administrative records

*Abbreviations: ANZDATA*, Australia New Zealand Dialysis and Transplant Registry; *EQ-5D-5L*, EuroQoL 5-item 5-level preference-based measure of health status; *VAS*, visual analogue scale; *SONG-HD Fatigue*, Standardised Outcomes in Nephrology – Haemodialysis, Fatigue; *IPOS Renal*, Integrated Palliative Outcome Scale – Renal; *Kt/V*, K=dialyser clearance of urea, t=dialysis time, V=volume of distribution of urea, this is approximately equal to the patient’s total body water

Participants in both the control and intervention arms are asked to complete the EQ-5D-5L and Standardised Outcomes in Nephrology-Haemodialysis (SONG-HD) Fatigue measure at baseline, 6 months and 12 months, as well as two fidelity questions regarding whether they discussed any symptoms with their treating health professionals (e.g., dialysis nurse, nephrologist, dietitian, social worker) at their last visit.

EQ-5D-5L, SONG-HD Fatigue measure and the IPOS-Renal responses will be collected from participants during their haemodialysis sessions. They will be completed electronically on an Android tablet provided to the centres by the trial. The data will be stored in Qualtrics (Qualtrics, Utah, 2021) survey platform, securely transferred daily to the SWIFT module within the ANZDATA Registry. Health-related QOL for both groups will be measured using the EQ-5D-5L (5 domains assessing mobility, self-care, usual activities, pain/discomfort, anxiety/depression), each with 5 levels of severity, and the visual analogue scale (VAS) on a 0 to 100 scale (0=worst imaginable health, 100= best imaginable health). QOL will be self-reported at baseline, 6 and 12 months. The EQ-5D-5L takes approximately 3 min to complete and is the most widely used and validated preference-based health-related QOL measure among dialysis patients [17]. It is sensitive to poor health states and high symptom burden associated with haemodialysis treatment [26]. The EQ-5D-5L is the measure that achieves the highest amount of complete data, making it the preferred choice for repeated measures collected within a registry [9]. SONG-HD Fatigue is a core outcome measure in haemodialysis trials and will be assessed using a 3-item questionnaire established by the Standardised Outcomes in Nephrology (SONG) Initiative that has been validated in the haemodialysis population [27]. Permission has been obtained for the IPOS Renal (Kings’ College, London, UK), EQ-5D-5L (EuroQol, Rotterdam, Netherlands) and SONG-HD Fatigue (SONG Initiative, Sydney Australia) to use these instruments in SWIFT via electronic data capture.

### Participant timeline {13}

Participant timeline including recruitment, intervention and outcome measures are displayed in Table 1.

### Sample size {14}

SWIFT will recruit 143 haemodialysis centres (or clusters) in Australia. Recruitment data from the SWIFT pilot study confirmed the assumptions of over 50% patient participation in each centre, and 80% completion of EQ-5D-5L at 12 months [17]. Sites are grouped into clusters (see 16a) of approximately 2 sites/cluster. Approximately seventy-one clusters in each arm (equating to 2422 total participants) will enable us to identify a clinically significant mean (0.07) 7% increase in health-related quality of life (from 0.59 [59%] to 0.66 [66%]) with 90% power. This minimum clinically important difference was determined through review of EQ-5D-5L studies that showed a change in overall utility of between 0.03 and 0.07 across a range of chronic conditions was associated with meaningful and observable improvements in QOL. This minimum effect of 0.07 was considered feasible and is supported by an observational study in New South Wales (NSW) that demonstrated improvement in EQ-5D-5L utility of 0.11 could be achieved with a focus on symptom management (internal publication). The power calculation is based on a two-sided *t*-test with alpha=0.05 and assumes a modest intra-cluster correlation coefficient of 0.1 and a design effect of 3.5 (to account for clustering, allowing for uneven cluster sizes) creating an effective sample size of 692. We assume a standard deviation (SD) of the change between baseline and 12 months of 0.281. The 7% represents a conservative estimate in effect however one which demonstrates a clinically meaningful improvement in QOL and translates to a societal benefit of 640 quality-adjusted life years (QALYs) per year.

### Recruitment {15}

Contact details of all potential dialysis centres have been sourced from the ANZDATA registry. Centres are invited via email to complete a pre-trial feasibility survey, exploring existing PROMs use and symptom management strategies. Eligible centres that agree to participate will be randomised. All patients attending that site over a 2-week period are invited to participate in the trial. New patients who may be in their first year of dialysis and who are not yet registered with the ANZDATA registry can be added to the Registry (and trial) before the baseline data collection commences. The ANZDATA Registry operates under an “opt out” model of consent; therefore, if a patient is in a participating centre and chooses not to be a part of the Registry, they are not able to join the trial as secondary outcomes will not be able to be collected for them.

Each patient will be provided with a Participant Information Sheet (PIS) and the opportunity to read the document and ask questions. The PIS was prepared with input from an ANZDATA consumer representative, Mr Shyamsundar Muthuramalingam (SM), and trialled by patients in the Royal Adelaide Hospital haemodialysis unit. Both the PIS and the PIS summary have been translated into the seven language translations used for SWIFT and are presented to patients in the unit. A screening log records any patients who decline participation in SWIFT and representativeness of the included sample will be reported. At the participant’s next dialysis session (at least 2 days later), the participant is asked to complete the online survey(s) specific to their trial allocation (intervention or control). The consent form is embedded into the baseline survey of the trial and will be completed electronically. Participants in both groups will be informed to raise any health concerns with their doctor or nurse.

### Assignment of interventions: allocation

#### Sequence generation {16a}

ANZDATA centres (i.e. clusters) will be randomised to intervention or control. The randomisation takes place, on a state-by-state basis using the method of minimisation as they agree to participate. This occurs once the SWIFT team has received the feasibility forms for all eligible centres in a state and confirmed which sites will participate. Centres (clusters) may consist of more than one site if frequent patient movement or otherwise close institutional linkages between sites prohibit distinction into multiple functionally distinct clusters. The randomisation will be stratified based upon location (state), metropolitan or regional, private or public unit or prior or current use of the IPOS-Renal questionnaire for symptom monitoring within each centre and cluster size. This will diminish potential confounding by these factors.

#### Concealment mechanism {16b}

Once participating sites are confirmed, the allocation process will be undertaken by the trial statistician (CB) and kept concealed until the site initiation visit. Access to the allocations is limited to the CI (RLM), the trial statistician (CB), the CTC trial operations coordinator (PW) and ANZDATA Registry Manager (Ms. Kylie Hurst) to minimise risk of inadvertently influencing sites or prematurely revealing allocation.

#### Implementation {16c}

Assignment to the intervention or control arm will be undertaken by the randomisation team at the National Health and Medical Research Council Clinical Trials Centre (NHMRC CTC). A customised randomisation system has been created for SWIFT. Clusters are sorted by stratification factors, paired, then arm allocation within pairs is determined by random selection (using the current date as a seed).

### Assignment of interventions: blinding

#### Who will be blinded {17a}

Blinding to allocation is not possible within clusters due to the nature of the intervention; however, all staff compiling and analysing outcome data will be blinded to allocation.

#### Procedure for unblinding if needed {17b}

Not applicable as intervention blinding is not possible.

### Data collection and management

#### Plans for assessment and collection of outcomes {18a}

Data is collected electronically via an Android tablet (five timepoints for the intervention arm and three timepoints for the control arm) and stored in the Qualtrics survey platform (Qualtrics, Utah, 2021). Each individual participant has a unique quick response (QR) code assigned to them for the duration of the trial that matches a unique “SWIFT ID”. Participant responses are automatically downloaded from the Qualtrics survey platform every 15 min (see data management statement below). The response date is automatically marked off for all survey responses. A manual query is run by LMG daily to determine if there is a duplicate response or if the incorrect QR code is scanned for a participant so the response can be queried with the centre. ANZDATA also receives a notification via email with the participant’s SWIFT ID, date of survey completion and the survey response for each participant as an additional check.

#### Plans to promote participant retention and complete follow-up {18b}

The SWIFT Pilot Study [17] determined that an important facilitator to the uptake and engagement in the trial was a nurse champion in each centre. These nurse champions influenced the rates of recruitment within each haemodialysis centre. Participant retention is potentially high given that the participants are attending their haemodialysis centre three times per week, unless they receive a transplant, die, stop haemodialysis or are transferred to another non-participating haemodialysis centre. Additionally, consumer involvement in the design of the trial was to aid feasibility. Symptoms are 3 of the top ten patient research priorities [28]. Dialysis patients and their family members have helped select the most meaningful and feasible QOL instruments.

#### Data management {6}

The data is stored in the Qualtrics (Qualtrics, Utah, 2021) survey platform and then securely transferred daily to the SWIFT module within the ANZDATA Registry. Data is transferred through use of an application programming interface (API) developed between ANZDATA and Qualtrics. Data will be stored in tables within the SWIFT ANZDATA module until analysis.

#### Confidentiality {27}

All data generated in this trial will remain confidential. All information will be stored securely at the NHMRC CTC, University of Sydney and ANZDATA (located within the South Australian Health and Medical Research Institute [SAHMRI]) and will only be available to trial investigators undertaking data cleaning and analysis. Personal data identifying trial participants will be held securely at the NHMRC CTC and ANZDATA for the purpose of data matching with the Health Insurance Commission (HIC). HIC approvals will be obtained.

#### Plans for collection, laboratory evaluation and storage of biological specimens for genetic or molecular analysis in this trial/future use {33}

Not applicable, no biological samples will be collected.

### Statistical methods

#### Statistical methods for primary and secondary outcomes {20a}

The primary analysis will compare symptom monitoring with feedback versus usual care to evaluate the effect of this intervention on QOL and secondary outcomes at 12 months. Change in EQ-5D-5L (from baseline) will be evaluated at 12 months using a generalised estimation equation regression model, adjusted for stratification factors and accounting for clustering. Analysis of outcomes will be compared using an intention-to-treat analysis and individual participant data will be used for all analyses. Sensitivity analyses excluding clusters that currently use IPOS-Renal as a part of their usual care and excluding participants actively managed through a kidney supportive care programme will be undertaken.

##### Economic analyses

Cost-effectiveness of symptom monitoring with feedback and QOL collection through the ANZDATA registry, versus no symptom monitoring or feedback, will be calculated from the perspective of the health system. In this within-trial analysis, measured costs will include the electronic (e)-PROMs platform for collection and feedback, the additional staff time to facilitate collection at each centre and receive, interpret and act upon symptom scores, dialysis modality costs, hospitalisations, Medical Benefits Scheme (MBS) and Pharmaceutical Benefits Scheme (PBS) claims, and the central coordinating centre staff time for analysis and incorporation of PROMs into ANZDATA annual reports. Benefits will be measured in quality-adjusted survival at 6 and 12 months, using values from the EQ-5D-5L questionnaire at 2 or 3 time points. A sensitivity analysis that includes all randomised patients will be undertaken to ascertain informative censoring from dropout as a competing risk. Results will be presented in disaggregated format for each group and combined into an incremental cost-effectiveness ratio. The cost per quality-adjusted life year (QALY) for each comparison will be compared to the Australian Government’s willingness to pay for QALY gains [29]. Uncertainty will be assessed through sensitivity analyses (using non-parametric bootstrapping) to test the robustness of results [30]. The economic evaluation will be reported according to the CHEERS statement [19]. The results will inform policy makers as to the cost-effectiveness of a novel “health services” intervention and ongoing financing of registry-based PROMs.

#### Interim analyses {21b}

No Interim analysis will be performed.

#### Methods for additional analyses (e.g. subgroup analyses) {20b}

Four secondary analyses are planned to evaluate early effects on QOL (after 6 months) for consistency with 12-month results, to evaluate relationships between symptoms and EQ-5D-5L in the intervention group, to assess the association between unit characteristics and changes in QOL and to determine the association between renal centre factors (size, public/private, metropolitan/rural), and changes in QOL.

#### Methods in analysis to handle protocol non-adherence and any statistical methods to handle missing data {20c}

Sensitivity analyses using multiple imputation methods will be considered to account for missing QOL data where participants are known to be alive, in accordance with the EQ-5D-5L user guide. Further details will be outlined in the statistical analysis plan.

#### Plans to give access to the full protocol, participant-level data and statistical code {31c}

The SWIFT full protocol, de-identified centre-level dataset and statistical code will be available on request following trial publication.

### Oversight and monitoring

#### Composition of the coordinating centre and trial steering committee {5d}

The SWIFT Trial Management Committee (TMC) will oversee trial planning, monitoring, progress, review of information from related research and implementation of recommendations from other trial committees and external bodies (e.g. ethics committees). A Trial Operations (Executive) group will be formed from the TMC to deal with day-to-day operational concerns. TMC meetings will occur weekly during recruiting years, with trial operations meeting quarterly. The TMC will consider whether to continue the trial as planned, modify, or stop it, based on or other information.

#### Composition of the data monitoring committee, its role and reporting structure {21a}

Data from this trial will be monitored by the Clinical Trials Program staff from the NHMRC CTC and ANZDATA (SWIFT team). Monitoring will include centralised review of electronic case report forms (eCRFs) and other trial documents for protocol compliance, data accuracy and completeness. Monitoring may include monitoring visits to investigational sites during the trial for source data verification and review of the investigator’s site file. The delegated staff will be given direct access to source documents, eCRFs and other trial-related documents. As detailed in the Participant Information Sheet and Consent Form, the participant gives authorised staff direct access to their medical records and the trial data. SWIFT is a low-risk trial with no drug or device being tested, therefore a specific, formal data monitoring committee is not required.

#### Adverse event reporting and harms {22}

Adverse events are not expected given the nature of the intervention. All participants will be encouraged to advise their clinical team if issues raised in the trial have caused them discomfort or distress. Participants are under the clinical care of their primary physician with no direct care provided by the trial team or sponsor.

#### Frequency and plans for auditing trial conduct {23}

All trial data collected via e-PROMs for this trial will be stored in a trial database (Qualtrics) and linked to the ANZDATA registry, thus capitalising on an established and secure data collection system. SWIFT will utilise linked data for health system resource use and costs, and routine ANZDATA registry outcomes for secondary endpoints. All trial data required for the monitoring and analysis of the trial will be recorded electronically on the case report forms (i.e. Qualtrics survey) provided. Source documents (e.g. dialysis treatment records) will be maintained by sites and may include a participant’s medical records, hospital charts and clinic charts. All trial-related documentation at sites will be maintained for 15 years following trial completion.

#### Plans for communicating important protocol amendments to relevant parties (e.g. trial participants, ethical committees) {25}

Any protocol amendments will be submitted to CALHN HREC prior to change of protocol. Once approved, participating centres will be notified immediately by the TMC via email of any amendments to the trial protocol.

### Dissemination plans {31a}

Results will be disseminated to the Australian Quality and Safety in Healthcare Commission, Federal and State Health departments and ministers, consumers through Kidney Health Australia (KHA), and Better Evidence and Translation in Chronic Kidney Disease (BEAT-CKD), ANZDATA Advisory Group, ANZDATA Registry funders including the OTA, NHMRC reporting mechanisms, International Society of Nephrology, Australia New Zealand Society of Nephrology (ANZSN) and Renal Society of Australasia (RSA) and through peer reviewed medical, nursing and health services publications, conference presentations and social media via #SWIFTtrial and @SWIFTtrial.

## Discussion

SWIFT is the first registry-based trial in the Australian haemodialysis population investigating whether regular symptom monitoring with feedback to participants and clinicians assists in improving QOL. This is significant because people receiving haemodialysis experience a multitude of symptoms related to their treatment [31] which in turn is associated with poorer quality of life [23].

We have previously demonstrated the feasibility of this study confirming that 72% of Australian and New Zealand haemodialysis centres indicated that they wished to participate in SWIFT [17]. This high rate of participation is likely to be associated with recognition of the importance of PROMs and ability of a registry-based trial to be inclusive of all centres regardless of whether they are a small rural facility, a large metropolitan hospital or a private dialysis centre.

The significance of SWIFT as an ANZDATA Registry embedded trial will facilitate the registry to expand on the outcomes collected to include PROMs if the trial determines this to be beneficial. This will complement haemodialysis services that currently have existing infrastructure of regional, national and international registries for collecting and reporting information on all patients receiving treatment (see Fig. 2) [32].

A limitation of SWIFT is the dependence on the clinician engagement with both participants receiving haemodialysis, and other health care professionals, such as dietitians, social workers and pharmacists once the symptom feedback is delivered to the nurse unit manager and nephrologist. Additionally, patients in the intervention arm who do not have access to email may not be able to access the feedback. Feedback emails can be sent to the email address of a nominated family member or friend or be sent to the generic email of the centre where it can be printed for the patients. In the centres where emails can be sent and oriented in the centre, this adds extra time involvement to an already busy centre. Not all patients will have access to these options which could make it harder to obtain the feedback. The major strength of this trial is the ability to report PROMs alongside standard clinical and biochemical measures in a clinical quality registry. Secondly, trials often are exclusive of non-English participants; therefore, another strength of this trial is that materials have been translated in to 7 languages other than English. This will ensure the trial’s ability to include a broader number of participants and thus ensure better representation of some of Australia’s diverse cultures.

By measuring and determining the effect of collecting and the feeding back of PROMs, SWIFT will draw attention and raise awareness of symptom burden and quality of life, which should drive research and quality improvement efforts in this area. Additionally, SWIFT will increase PROMs as a priority for service investment and improvement with the ultimate aim of measuring what is important to the people that matter—those living with kidney failure.

## Trial status

Protocol version 3 date: 14 December 2020

Recruitment start date: 7 April 2021

Recruitment end date: 7 September 2023

## Supplementary Information

**Additional file 1.** .

## Abbreviations

ANZDATA: Australia and New Zealand Dialysis and Transplant Registry
CTC: NHMRC Clinical Trials Centre, University of Sydney
EQ-5D-5L: EuroQoL 5-item 5-level preference-based measure of health status
HREC: Human Research Ethics Committee
IPOS Renal: Integrated Palliative Outcome Scale – Renal
MBS: Medicare Benefits Schedule (Australia)
PBS: Pharmaceutical Benefits Scheme (Australia)
PROMs: Patient Reported Outcome Measures
QALY: Quality-adjusted life year
QOL: Quality of life
SAHMRI: South Australian Health and Medical Research Institute
SONG-HD Fatigue: Standardised Outcomes in Nephrology – Haemodialysis, Fatigue
SPIRIT: Standard Protocol Items: Recommendations for Interventional Trials
TMC: Trial Management Committee
